# Two new species of *Microdochium* from *Indocalamus longiauritus* in south-western China

**DOI:** 10.3897/mycokeys.72.55445

**Published:** 2020-09-10

**Authors:** Shengting Huang, Jiwen Xia, Xiuguo Zhang, Wenxiu Sun, Zhuang Li

**Affiliations:** 1 College of Life Science, Yangtze University, Jingzhou, Hubei 434025, China Yangtze University Jingzhou China; 2 Shandong Provincial Key Laboratory for Biology of Vegetable Diseases and Insect Pests, College of Plant Protection, Shandong Agricultural University, Taian, 271018, China Shandong Agricultural University Taian China

**Keywords:** Microdochiaceae, multigene phylogeny, new species, taxonomy, Xylariales

## Abstract

*Microdochium* species have often been reported as plant pathogens and saprophytes and are commonly isolated from some diseased plant hosts. The primary aim of the present study was to describe and illustrate two new *Microdochium* species isolated from the leaf spot of *Indocalamus longiauritus* in Yunnan Province, China, namely *Microdochium yunnanense* and *M. indocalami*, spp. nov., based on their morphology and multilocus phylogenetic analyses of the combined ITS, LSU, TUB2, and RPB2. DNA sequence data indicate that six strains represent three independent groups from related and similar species in *Microdochium*. *Microdochium indocalami***sp. nov.** clustered with *M. fisheri*, *M. lycopodinum*, *M. rhopalostylidis*, and *M. phragmitis*. *Microdochium yunnanense***sp. nov.** grouped with *M. bolleyi*. In addition, the strain SAUCC1017 is recorded as an unidentified species in *Microdochium*. Descriptions and illustrations of the new species in the genus and *Microdochium* sp. indet. are provided.

## Introduction

*Microdochium* is a genus in Microdochiaceae (Xylariales), which has been well-studied in recent years by Hernández-Restrepo et al. (2016), Zhang et al. (2017), Crous et al. (2018, 2019), and Marin-Felix et al. (2019) by incorporating morphological and molecular data with appropriate genes to resolve species limitations in the genus. *Microdochium phragmitis*, the type of the genus, was introduced by Sydow (1924) for an ascomycetous fungal plant pathogen found on leaves of *Phragmites australis* (= *Phragmites communis*) in Germany in 1919, which has globose, erumpent stromata of minute, hyaline cells, small papillate conoid conidiogenous cells, and solitary, fusiform to subfalcate hyaline conidia. Currently, about 48 species of this genus are listed in Index Fungorum (http://www.indexfungorum.org/; accessed 1 May 2020), but only about two-fifths of them are well known and have been studied in pure culture (Crous et al. 2018, 2019; Marin-Felix et al. 2019).

*Monographella* was described by Petrak (1924) and was considered the sexual morph of *Microdochium* for many years (Hernández-Restrepo et al. 2016). Nevertheless, *Microdochium* has more species, is more commonly encountered, and the name is more frequently used in the literature. With the implementation of “one fungus one name” nomenclature, *Microdochium* has been retained as a genus name (Hernández-Restrepo et al. 2016).

*Microdochium* included important plant pathogens, particularly on grasses and cereals. Kwasna et al. (2007) newly described *M. triticicola* which was isolated from roots of wheat in the United Kingdom. Zhang et al. (2015) identified *M. paspali*, which caused leaf blight of seashore paspalum (*Paspalum vaginatum*), a turfgrass widely used in tropical and subtropical golf courses. Crous et al. (2018) described *M. musae* isolated from leaves of *Musa* sp. *Microdochium rhopalostylidis*, found on the leaves of *Rhopalostylis sapida* (Arecaceae) in New Zealand, was identified and described by Crous et al. (2019). From turf leaves (Poaceae) in New Zealand, *M. novae-zelandiae* was isolated by Marin-Felix et al. (2019) and described as a new species.

Many taxonomic problems have occurred in *Microdochium*, and the genus was shown to be polyphyletic (Hernández-Restrepo et al. 2016). However, some species have been reclassified based on molecular analyses (Glynn et al. 2005; Jewell and Hsiang 2013; Hernández-Restrepo et al. 2016). For example, phylogenetic analysis of the translation elongation factor 1-alpha gene (TEF1) showed that the isolates previously described as varieties of *Microdochium nivale* shown a distinct heterogeneity between isolates and these isolates were generated as two separate species, *M. majus* and *M. nivale* (Glynn et al. 2005). The study of multigene differences between *M. nivale* and *M. majus* by Jewell and Hsiang (2013) supported the reclassification of *M. nivale* and *M. majus* as sister species rather than varieties. Three species of *Microdochium* were revised by Hernández-Restrepo et al. (2016), which were initially recognised as *M. gracile* (CBS 493.70), *M. tripsaci* (CBS 857.72), and *M. fusarioides* (CBS 740.83, CBS 741.83, and CBS 742.83), and were renamed *Paramicrodochium gracile* (Sordariomycetes*incertae sedis*), *Ephelis tripsaci* (Clavicipitaceae, Hypocreales), and *Microdochiella fusarioides* (Orbiliales).

In this study, we introduce two novel species, *M. yunnanense* and *M. indocalami* spp. nov., which were isolated from the leaves of *Indocalamus longiauritus* in China. These two species are introduced based on both morphological features and molecular sequence data.

## Materials and methods

### Isolation and morphological studies

The samples were collected from Yunnan Province, China. The strains of *Microdochium* were isolated from diseased or healthy leaves of *Indocalamus longiauritus* using single spore and tissue isolation methods (Chomnunti et al. 2014). Single spore isolation following the protocol of Choi et al. (1999) and Zhang et al. (2013) was adopted for collection with visible foliar sporulation. The spore suspension was obtained and spread onto potato dextrose agar (PDA) and incubated overnight under normal conditions. The germinated spores were then transferred to a new PDA plate to obtain a pure culture. Besides, the surface-sterilised plant tissue isolation was also used to obtain sterile isolates from plant host. Fungi were isolated by cutting eight fragments (5 × 5 mm) per leaf from the margin of leaf lesions and surface-sterilized by consecutively immersing in 75% ethanol solution for 1 min, 5% sodium hypochlorite solution for 30 s, and then rinsing in sterile distilled water for 1 min (Gao et al. 2014; Liu et al. 2015). The samples were dried with sterilized paper towels and placed on potato dextrose agar (PDA) (Cai et al. 2009). All the plates were incubated at biochemical incubator at 25 °C for 3–4 days, then hyphae were picked out of the periphery of the colonies and inoculated onto new PDA plates.

Following 2–3 weeks of incubation, morphological characters were recorded as by Hernández-Restrepo et al. (2016). Photographs of the colonies were taken at 7 days and 15 days using a Powershot G7X mark II digital camera. Micromorphological characters were observed using an Olympus SZX10 stereomicroscope and an Olympus BX53 microscope, both fitted with Olympus DP80 high definition color digital cameras to photo-document fungal structures. All fungal strains were stored in 10% sterilized glycerin at 4 °C for further studies. Voucher specimens were deposited in the Herbarium of the Department of Plant Pathology, Shandong Agricultural University (HSAUP). Living cultures were deposited in the Shandong Agricultural University Culture Collection (SAUCC). Taxonomic information of the new taxa was submitted to MycoBank (http://www.mycobank.org).

### DNA extraction and amplification

Genomic DNA was extracted from fungal mycelia grown on PDA, using a modified cetyltrimethylammonium bromide (CTAB) protocol as described in Guo et al. (2000). Four pairs of primers were adopted to amplify gene sequences (Hernández-Restrepo et al. 2016). The partial large subunit (LSU) rDNA, the internal transcribed spacer region with intervening 5.8S nrRNA gene (ITS), part of the beta-tubulin gene region (TUB2), and partial RNA polymerase II second largest subunit (RPB2) region were amplified and sequenced using primers pairs LR0R/LR5 (Vilgalys and Hester 1990), ITS4/ITS5 (White et al. 1990), Btub526F and Btub1332R (Jewell and Hsiang 2013), and RPB2-5F2/fRPB2-7cR (Liu et al. 1999; Sung et al. 2007), respectively.

PCR was performed using an Eppendorf Master Thermocycler (Hamburg, Germany). Amplification reactions were performed in a 25 μL reaction volume, which contained 12.5 μL Green Taq Mix (vazyme, Nanjing, China), 1 μL of each forward and reverse primer (10 μM) (Biosune, Shanghai, China), and 1 μL template genomic DNA in amplifier, and were adjusted with distilled deionized water to a total volume of 25 μL.

PCR parameters were as follows: 94 °C for 5 min, followed by 35 cycles of denaturation at 94 °C for 30 s, annealing at a suitable temperature for 30 s, extension at 72 °C for 1 min and a final elongation step at 72 °C for 10 min. Annealing temperature for each gene were 55 °C for ITS, 51 °C for LSU, 56 °C for RPB2 and 53 °C for TUB2. The PCR products were visualised on 1% agarose electrophoresis gel. Sequencing was done bi-directionally, conducted by the Biosune Company Limited (Shanghai, China). Consensus sequences were obtained using MEGA 7.0 (Kumar et al. 2016). All sequences generated in this study were deposited in GenBank (Table 1).

### Phylogenetic analyses

Novel sequences generated from the six strains in this study, and all reference available sequences of *Microdochium* species downloaded from GenBank (mostly used in Hernández-Restrepo et al. 2016; Zhang et al. 2017; Marin-Felix et al. 2019; Crous et al. 2018, 2019) were used for phylogenetic analyses. Alignments of the individual locus were determined using MAFFT v. 7.110 by default settings (Katoh et al. 2017) and manually corrected where necessary. To establish the identity of the isolates at species level, phylogenetic analyses were conducted first individually for each locus and then as combined analyses of four loci (ITS, LSU, TUB2, and RPB2 regions). Phylogenetic analyses were based on maximum likelihood (ML) and Bayesian inference (BI) for the multi-locus analyses. For BI, the best evolutionary model for each partition was determined using MrModeltest v. 2.3 (Nylander 2004) and incorporated into the analyses. ML and BI were run on the CIPRES Science Gateway portal (https://www.phylo.org/) (Miller et al. 2012) using RaxML-HPC2 on XSEDE (8.2.12) (Stamatakis 2014) and MrBayes on XSEDE (3.2.7a) (Huelsenbeck and Ronquist 2001; Ronquist and Huelsenbeck 2003; Ronquist et al. 2012), respectively. For ML analyses the default parameters were used and BI was carried out using the rapid bootstrapping algorithm with the automatic halt option. Bayesian analyses included four parallel runs of 5,000,000 generations, with the stop rule option and a sampling frequency of 500 generations. The burn-in fraction was set to 0.25 and posterior probabilities (PP) were determined from the remaining trees. The resulting trees were plotted using FigTree v. 1.4.2 (http://tree.bio.ed.ac.uk/software/figtree) and edited with Adobe Illustrator CS5.1. New sequences generated in this study were deposited at GenBank (https://www.ncbi.nlm.nih.gov; Table 1), the alignments and trees were deposited in TreeBASE (http://treebase.org/treebase-web/home.html).

**Table 1.** Specimens and GenBank accession numbers of DNA sequences used in this study.

Species	Voucher	Host/Substrate	Country	GeneBank accession numbers			
				LSU	ITS	TUB2	RPB2
*Idriella lunata*	CBS 204.56*	Root of *Fragaria chiloensis*	USA	KP858981	KP859044	–	–
*Microdochium albescens*	CBS 291.79	On *Oryza sativa*	Ivory Coast	KP858932	KP858996	KP859059	KP859105
	CBS 243.83	Seed *Oryza sativa*	Unknown country	KP858930	KP858994	KP859057	KP859103
*M. bolleyi*	CBS 540.92	Root of *Hordeum vulgare*	Syria	KP858946	KP859010	KP859073	KP859119
*M. citrinidiscum*	CBS 109067*	Leaf of *Eichhornia crassipes*	Peru	KP858939	KP859003	KP859066	KP859112
*M. chrysanthemoides*	LC5363 = CGMCC3.17929*	Unnamed Karst Cave	China	KU746736	KU746690	KU746781	–
	LC5466 = CGMCC3.17930	Unnamed Karst Cave	China	KU746735	KU746689	KU746782	–
*M. colombiense*	CBS 624.94*	On *Musa sapientum*	Colombia	KP858935	KP858999	KP859062	KP859108
*M. fisheri*	CBS 242.90*	Stem of *Oryza sativa*	UK	KP858951	KP859015	KP859079	KP859124
***M. indocalami***	SAUCC1016*	Leaves of *Indocalamus longiauritus*	China	MT199878	MT199884	MT435653	MT510550
*M. lycopodinum*	CBS 146.68	Air sample	The Netherlands	KP858929	KP858993	KP859056	KP859102
	CBS 109397	On *Phragmites australis*	Germany	KP858940	KP859004	KP859067	KP859113
	CBS 109398	On *Phragmites australis*	Germany	KP858941	KP859005	KP859068	KP859114
*M. majus*	CBS 741.79	On *Triticum aestivum*	Germany	KP858937	KP859001	KP859064	KP859110
*M. musae*	CBS 111018 = CPC 5380	*Musa* cv. Cavendish	Costa Rica	–	AY293061	–	–
	CBS 143499 = CPC 32809	Leaves of *Musa* sp.	Malaysia	MH107941	MH107894	MH108040	–
	CBS 143500* = CPC 32689	Leaves of *Musa* sp.	Malaysia	MH107942	MH107895	MH108041	MH108003
	CPC 11234	Leaves of *Musa* sp.	Mauritius	MH107943	MH107896	MH108042	–
	CPC 11240	Leaves of *Musa* sp.	Mauritius	MH107944	MH107897	MH108043	–
	CPC 16258	Leaves of *Musa* sp.	Mexico	MH107945	MH107898	MH108044	–
	CPC 32681	Leaves of *Musa* sp.	Malaysia	MH107946	MH107899	–	–
*M. neoqueenslandicum*	CBS 445.95	On *Juncus effusus*	The Netherlands	KP858933	KP858997	KP859060	KP859106
	CBS 108926*	On *Agrostis* sp.	New Zealand	KP858938	KP859002	KP859065	KP859111
*M. nivale*	CBS 116205*	Roots of *Triticum aestivum*	UK	KP858944	KP859008	KP859071	KP859117
M. nivale var. nivale	CBS 288.50	Unknown	Unknown country	MH868135	MH856626	–	–
*M. novae-zelandiae*	CBS 143847	From turf leaves (Poaceae)	New Zealand	–	LT990655	LT990608	LT990641
	CPC 29693	From turf leaves (Poaceae)	New Zealand	–	LT990656	LT990609	LT990642
*M. paspali*	HK-ML-1371	*Paspalum vaginatum*	China	–	KJ569509	KJ569514	–
	QH-BA-48	*Paspalum vaginatum*	China	–	KJ569510	KJ569515	–
	SY-LQG66	*Paspalum vaginatum*	China	–	KJ569511	KJ569516	–
	WC-WC-85	*Paspalum vaginatum*	China	–	KJ569512	KJ569517	–
	WN-BD-452	*Paspalum vaginatum*	China	–	KJ569513	KJ569518	–
*M. phragmitis*	CBS 285.71*	On *Phragmites australis*	Poland	KP858949	KP859013	KP859077	KP859122
	CBS 423.78	On *Phragmites communis*	Germany	KP858948	KP859012	KP859076	KP859121
*M. rhopalostylidis*	CPC 34449 = CBS 145125*	*Rhopalostylis sapida*	New Zealand	MK442532	MK442592	MK442735	MK442667
*M. seminicola*	KAS3576 = CBS 139951*	Maize kernels	Switzerland	KP858974	KP859038	KP859101	KP859147
	KAS1516 = CPC 26001	On grain	Canada	KP858961	KP859025	KP859088	KP859134
	KAS3574 = DAOM 250155	Maize kernels	Switzerland	KP858973	KP859037	KP859100	KP859146
	KAS3158 = DAOM 250161	On *Triticum aestivum*	Canada	KP858970	KP859034	KP859097	KP859143
	KAS1527 = DAOM 250165	On grain	Canada	KP858966	KP859030	KP859093	KP859139
	KAS1473 = DAOM 250176	On *Triticum aestivum*	Canada	KP858955	KP859019	KP859082	KP859128
*M. sorghi*	CBS 691.96	Living *Sorghum halepense*	Cuba	KP858936	KP859000	KP859063	KP859109
***M.* sp. indet.**	SAUCC1017	Leaves of *Indocalamus longiauritus*	China	MT199879	MT199885	MT435654	–
*M. tainanense*	CBS 269.76*	Root of *Saccharum officinarum*	China, Taiwan	KP858945	KP859009	KP859072	KP859118
	CBS 270.76	Root of *Saccharum officinarum*	China, Taiwan	KP858931	KP858995	KP859058	KP859104
*M. trichocladiopsis*	CBS 623.77*	Rhizosphere of *Triticum aestivum*	Unknown country	KP858934	KP858998	KP859061	KP859107
***M. yunnanense***	SAUCC1011*	Leaves of *Indocalamus longiauritus*	China	MT199875	MT199881	MT435650	MT510547
	SAUCC1012	Leaves of *Indocalamus longiauritus*	China	MT199876	MT199882	MT543651	MT510548
	SAUCC1015	Leaves of *Indocalamus longiauritus*	China	MT199877	MT199883	MT435652	MT510549
	SAUCC1018	Leaves of *Indocalamus longiauritus*	China	MT199880	MT199886	MT435655	–

Isolates marked with “*” are ex-type or ex-epitype strain.

## Results

### Phylogenetic analyses

**Figure 1.**
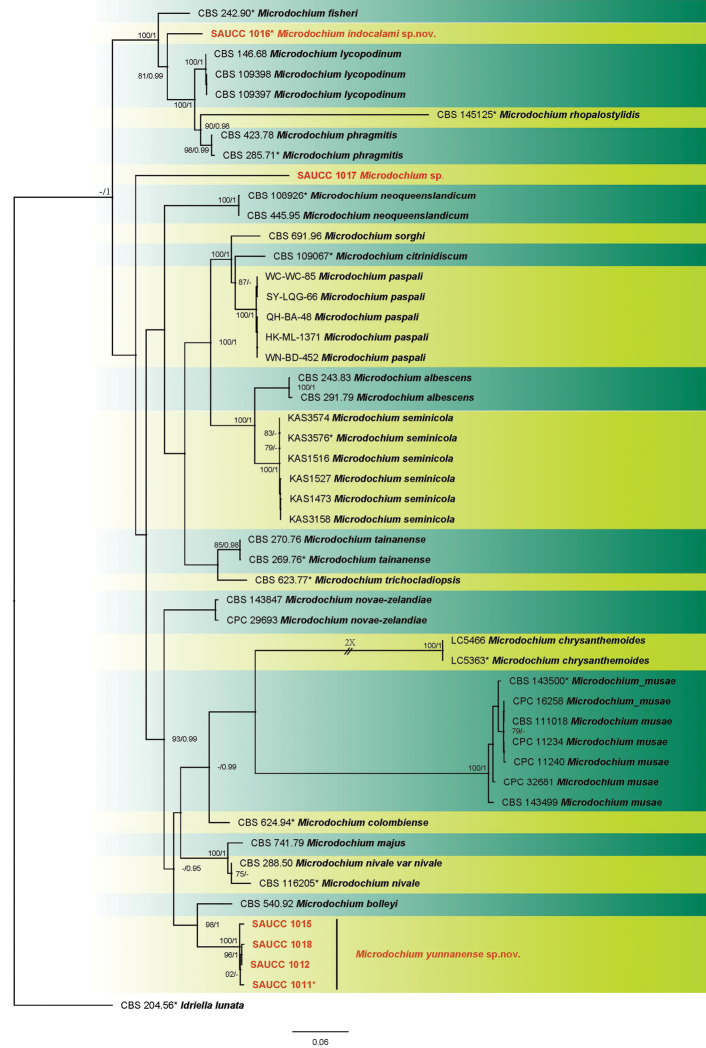
Phylogram of *Microdochium* based on combined ITS, LSU, TUB2 and RPB2 genes. The ML and BI bootstrap support values above 75% and 0.95 BYPP are shown at the first and second position, respectively. Strains marked with “*” are ex-type or ex-epitype. Strains from the current study are in red. Some branches were shortened to fit them to the page – these are indicated by two diagonal lines with the number of times a branch was shortened indicated next to the lines.

Six *Microdochium* strains isolated from plant hosts were sequenced. *Microdochium* was analysed by using multilocus data (ITS, LSU, TUB2 and RPB2) composed of 50 isolates of *Microdochium* and *Idriella lunata* (CBS 204.56) as the outgroup taxon. A total of 3257 characters including gaps were obtained in the phylogenetic analysis, viz. ITS: 1–572, LSU: 573–1429, TUB2: 1430–2395, RPB2: 2396–3257. Of these characters, 2019 were constant, 219 were variable and parsimony-uninformative, and 1019 were parsimony-informative. For the BI and ML analyses, GTR+I+G for LSU and RPB2, SYM+I+G for ITS, and GTR+G for TUB2 were selected and incorporated into the analyses. The ML tree topology confirmed the tree topologies obtained from the BI analyses, and therefore, only the ML tree is presented (Fig. 1).

ML bootstrap support values (≥ 75%) and Bayesian posterior probability (≥ 0.95) are shown as first and second position above nodes, respectively. The 50 strains were assigned to 23 species clades based on the four gene loci phylogeny (Fig. 1). The six strains studied here represented two novel species. The new species of *Microdochium yunnanense* showed a close relationship to *M. bolleyi* (CBS 540.92) with good support (ML-BS: 98% and BYPP: 1.00). *Microdochium indocalami* (SAUCC1016) appeared most closely related to *M. fisheri* (CBS 242.90), *M. lycopodinum* (CBS 146.68), *M. rhopalostylidis* (CBS 145125), and *M. phragmitis* (CBS 285.71) with high support by the multi-locus phylogeny. From the tree (Fig. 1), strain SAUCC1017 formed a conspicuous branch independent from other *Microdochium* species, thus supporting the introduction of SAUCC1017 as an indeterminate species.

### Taxonomy

#### Microdochium indocalami

Taxon classificationFungiXylarialesMicrodochiaceae

S.T. Huang, J.W. Xia, X.G. Zhang, W.X. Sun & Z. Li sp. nov.

C2015F60-88EF-51A8-9D84-260F88607CFB

835766

Figure 2

##### Etymology.

Name refers to the genus of the host plant *Indocalamus longiauritus*.

**Figure 2.**
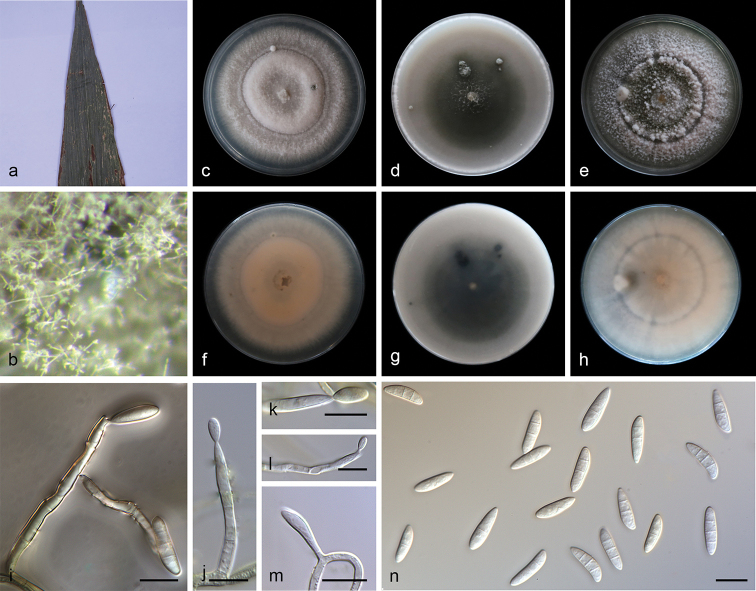
*Microdochium indocalami* (SAUCC1016) **a** leaves of host plant **b** colony overview **c–e** surface of colony after 15 days on PDA (**c**), OA (**d**), MEA (**e**) **f–h** reverse of colony after 15 days on PDA (**f**) OA (**g**) MEA (**h**) **i–m** conidiophores and conidiogenous cells **n** conidia. Scale bars: 10μm (**i–n**).

##### Diagnosis.

Characterised by the size of conidia and the number of septa of conidia.

##### Type.

China, Yunnan Province: Xishuangbanna Tropical Botanical Garden, Chinese Academy of Sciences, on diseased leaves of *Indocalamus longiauritus*. 16 April 2019, S.T. Huang, HSAUP1016, holotype, ex-type living culture SAUCC1016.

##### Description.

Colonies on PDA attaining 46.1–51.2 mm in diameter after 7 days, formed a conspicuous concentric circle, periphery of aerial mycelium cottony, centre with scarce aerial mycelium, white initially, then becoming greyish sepia after 25 days. Some aerial hyphae aggregated and form a sporodochium within 15 days or longer. Mycelium composed of hyaline, immersed and superficial, smooth, branched, septate, 2.0–3.0 μm wide hyphae. Due to the soluble pigment secreted, reverse white to salmon. Conidiophores straight or slightly curved, aseptate, aggregated in the aerial mycelium, often reduced to conidiogenous cells borne directly from the hyphae. Conidiogenous cells terminal or intercalary, mono-or polyblastic, denticulate, smooth, hyaline, cylindrical, straight or bent, 11.0–28.3 × 1.5–2.9 μm. Conidia cylindrical, clavate to obovoid, 1–3-septate, 13.0–15.5 × 3.5–5.5 μm, base usually flattened 0.5–1.0 μm. Sometimes borne directly from the mycelial hyphae. Sexual morph: unknown.

##### Culture characteristics.

Colonies on OA 62.0–64.0 mm in diameter after 7 days, centre with aerial mycelium cottony, periphery with scarce aerial mycelium. Mycelium mostly immersed, hyphae hyaline, septate, smooth, exudate and soluble pigment produced, reverse white initially, then becoming pale mouse-grey in periphery and mouse-grey in center. Sporodochia formed on agar surface. Colonies on MEA 50.8–52.7 mm in diameter after 7 days, aerial mycelium abundant, with concentric rings, white to pale pink, periphery with cottony aerial mycelium, centre with scarce aerial mycelium, exudate absent. Reverse white to pale pink with age.

##### Habitat and distribution.

Isolated from leaves of *Indocalamus longiauritus* in China.

##### Notes.

*Microdochium* and allied genera were revised by Hernández-Restrepo et al. (2016). Phylogenetic analysis of a combined four gene showed that *M. indocalami* (strain SAUCC1016) formed a separated branch as the clade of *M. fisheri*, *M. lycopodinum*, *M. phragmites*, and *M. rhopalostylidis* with good support (ML-BS: 100% and BYPP: 1.00) (Fig. 1). Additionally, conidiogenous cells of *M. indocalami* are terminal or intercalary, denticulate, cylindrical which are similar to the species in this clade. The size of conidia and the number of septa of conidia reported for *M. fisheri* (7.0–12.0 × 3.0–4.0 μm, 0–1-septate), *M. lycopodinum* (8.0–15.5 × 2.5–4.0 μm, 0–1-septate), *M. phragmites* (10.0–14.5 × 2.0–3.0 μm, 0–1-septate), and *M. rhopalostylidis* ((13.0–) 16.0–20.0 (–23.0) × (2.5–) 3.0 (–4.0) μm, 1–3-septate) (Hernández-Restrepo et al. 2016; Crous et al. 2019) were different to *M. indocalami* (13.0–15.5 × 3.5–5.5 μm, 1–3-septate).

#### Microdochium yunnanense

Taxon classificationFungiXylarialesMicrodochiaceae

S.T. Huang, J.W. Xia, X.G. Zhang, W.X. Sun & Z. Li sp. nov.

DA50595A-936A-5A65-AE44-C5BE833BC6D8

835765

Figure 3

##### Etymology.

Named after Yunnan Province, where the fungus was collected.

**Figure 3.**
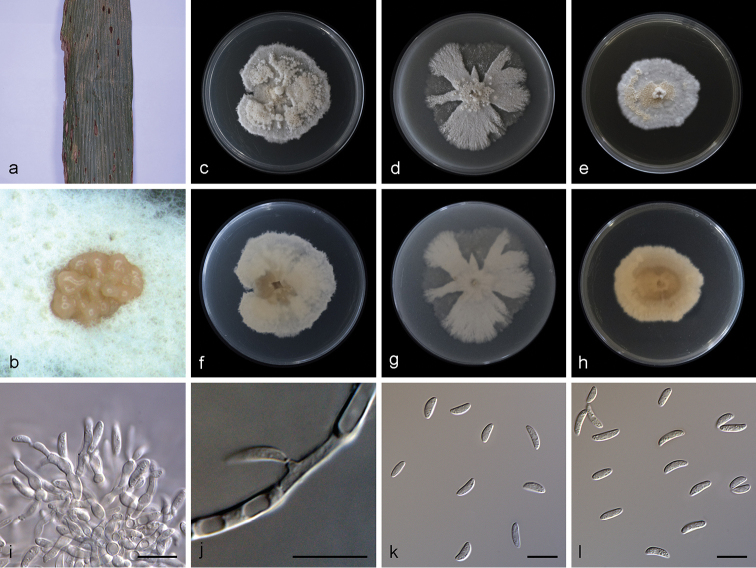
*Microdochium yunnanense* (SAUCC1011) **a** leaves of host plant **b** sporodochia on media surface **c–e** surface of colony after 15 days on PDA (**c**), OA (**d**), MEA (**e**) **f–h** reverse of colony after 15 days on PDA (**f**), OA (**g**), MEA (**h**) **i** sporodochial conidiophores and conidiogenous cells with conidia **j** conidiogenous cells **k–l** conidia. Scale bars: 10μm (**i–l**).

##### Diagnosis.

Characterised by conidiomata sporodochium-like and the size of conidia.

##### Type.

China, Yunnan Province: Xishuangbanna Tropical Botanical Garden, Chinese Academy of Sciences, on diseased leaves of *Indocalamus longiauritus*. 16 April 2019, S.T. Huang, HSAUP1011 holotype, ex-type living culture SAUCC1011.

##### Description.

Colonies on PDA attaining 48.0–61.5 mm in diameter after 15 days, felty, compact, erose or dentate, initially white, then becoming yellowish with age. Mycelium superficial, consisting of hyaline, smooth, branched, septate, 1.0–2.5 μm wide hyphae. Conidiomata sporodochium-like, appeared within 15 days or longer, formed in aerial mycelium or on agar surface, slimy, hyaline or orange, semi-submerged. Exudate occasionally appeared on old sporodochia. Reverse colorless to yellowish, due to the soluble pigment secreted. Conidiophores formed terminal or lateral with sympodial proliferation, solitary or aggregated. Most conidiophores tightly aggregated in a sporodochium, inconspicuous flat-tipped loci, irregularly branched, or borne directly on mycelial hyphae, straight or slightly curved, aseptate, guttulate, smooth-walled, apex subobtuse, base truncate. Conidiogenous cells of two types: some polyblastic, ampulliform, lageniform, with percurrent proliferations, 6.5–10.0 × 2.5–3.4 μm, neck up to 4.5 μm long, 1.0–1.5 μm wide, others straight or bent, smooth, cylindrical up to 10.0–11.5 μm long, 1.0–2.0 μm wide. Conidia aseptate, mostly lunate, a few ellipsoid and cylindrical, hyaline, straight or curved, obtuse, 0–2 guttulate in mature conidia, 6.8–10.0 × 2.4–3.5 μm. Chlamydospores was not observed. Sexual morph: unknown.

##### Culture characteristics.

Colonies on OA 58.1–61.5 mm in diameter after 15 days, entire, flat, white, lobate and radially margin, aerial mycelia cottony or sparse. Less exudate. Reverse white. Vegetative hyphae hyaline, abundant, branched, septate, thin-walled. Colonies on MEA 39.5–48.2 mm in diameter after 15 days, dense, initially white, becoming pale yellow, the centre of aerial mycelium cottony, periphery with scarce aerial mycelium, aerial mycelium formed a protuberance at center of colony.

##### Habitat and distribution.

Isolated from leaves of *Indocalamus longiauritus* in China.

##### Additional specimens examined.

China, Yunnan Province: Xishuangbanna Tropical Botanical Garden, Chinese Academy of Sciences, on diseased leaves of *Indocalamus longiauritus*. 16 April 2019, S.T. Huang, HSAUP1012, HSAUP1015, and HSAUP1018 paratype; living culture SAUCC1012, SAUCC1015, and SAUCC1018.

##### Notes.

Strains SAUCC1011, SAUCC1012, SAUCC1015, and SAUCC1018 belong to a single species as they have similar morphological features including culture characteristics, sporodochium, and conidia, the nearly identical sequence data, and cluster in a separate branch with a good support (ML-BS: 100% and BYPP: 1.00). The species is most phylogenetically close to *M. bolleyi*, and their branch lengths are slightly different. Nevertheless, the morphology of *M. yunnanense* and *M. bolleyi* (Hong et al. 2008) are different in having sporodochium-like conidiomata, conidiogenous cells, and conidia. *Microdochium yunnanense* produced some sporodochium-like conidiomata, slimy, hyaline or orange, semi-submerged on the agar surface, with most conidial droplets formed on it and a few formed laterally along with hyaline hyphal cells. However, the conidia of *M. bolleyi* only formed laterally along with hyaline hyphal cells. They all produced two types of conidiogenous cells, cylindrical and ampulliform, but the size of *M. yunnanense* (10.0–11.5 × 1.0–2.0 μm (average 10.7 × 1.6 µm) and 6.5–10.0 × 2.5–3.4 μm (average 8.4 × 2.9 µm)), *M. bolleyi* (1.5–2.7 × 0.8–1.4 µm (average 2.1 × 1.0 µm) and 3.1–6.4 × 2.5–3.8 µm (average 4.9 × 3.2 µm)) were clearly different. Conidial shape was differed little, but the conidial size of *M. yunnanense* (6.8–10.0 × 2.4–3.5μm (average 8.3 × 3.1 µm)) has much larger than *M. bolleyi* (5.0–8.7 × 1.6–2.3 µm (average 6.4 × 1.9 µm)).

#### Microdochium

Taxon classificationFungiXylarialesMicrodochiaceae

sp. indet.

3673BE11-371A-55F2-A66B-5B44B06C2F4D

Figure 4

##### Description.

Colonies on PDA attaining 73.9–80.4 mm in diameter after 15 days, felty to cottony, flat, margin entire or dentate, white, aerial mycelium abundant. Mycelium superficial, hyphae hyaline, septate, branched, smooth-walled. Reverse white to pale yellow, with yellow pigment produced with aging. Aerial hyphae aggregated to form numerous chlamydospores on agar surface. Chlamydospores thick-walled, terminal or intercalary, more frequently arranged in chains than clusters. Conidiophores not observed. Colonies on OA attaining 79.9–81.7 mm in diameter after 15 days, fluffy, margin entire, white. Reverse white. Colonies on MEA attaining 73.2–78.4 mm in diameter after 15 days, flat, with pale pink inconspicuous concentric circle near the centre, margin entire and white, aerial mycelium abundant.

**Figure 4.**
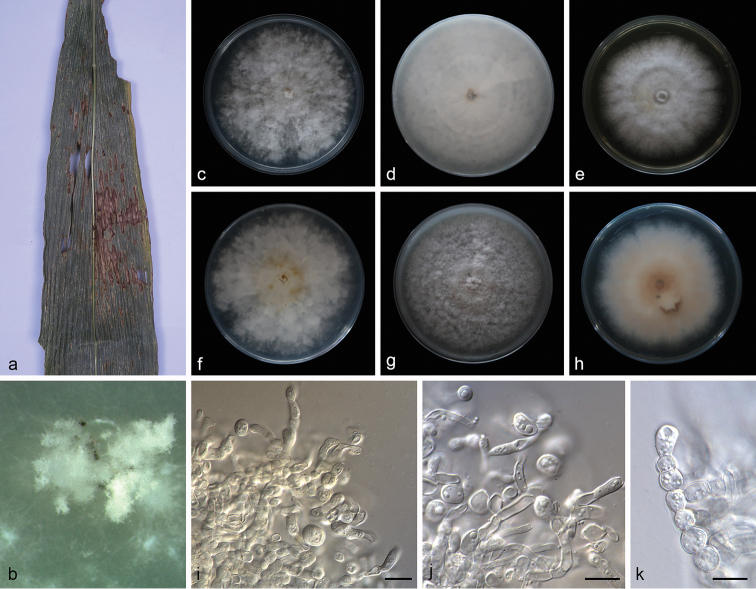
*Microdochium* sp. (SAUCC1017) **a** leaves of host plant **b** colony overview **c–e** surface of colony after 15 days on PDA (**c**) OA (**d**) MEA (**e**) **f–h** reverse of colony after 15 days on PDA (**f**) OA (**g**) MEA (**h**) **i–k** chlamydospores. Scale bars: 10μm (**i–k**).

##### Material examined.

China, Yunnan Province: Xishuangbanna Tropical Botanical Garden, Chinese Academy of Sciences, on diseased leaves of *Indocalamus longiauritus*. 16 April 2019, S.T. Huang, HSAUP1017, living culture SAUCC1017.

##### Note.

Strain SAUCC1017 failed to produce conidia and lacks a complete morphological description. It formed a conspicuous independent lineage from other *Microdochium* species in the tree. ITS sequence BLASTn search of SAUCC1017 showed many different species with 97% identity. BLASTn searches with LSU (GenBank MH869857) sequences result in 99% identity with *M. bolleyi* (CBS 172.63) and TUB2 (GenBank AB625368) sequences result in 99% identity with *Xylaria cubensis* (strain BCC 18758). Thus, here we listed it as an unidentified species.

## Discussion

Previous studies placed *Microdochium* in Amphisphaeriaceae (Parkinson et al. 1981; Samuels and Hallet 1983; von Arx 1984; Jaklitsch and Voglmayr 2012), which is a large heterogeneous family possessing pestalotiopsis-like asexual morphs characterised by holoblastic conidiogenous cells that produce septate, brown or hyaline conidia with appendages at both ends (Tanaka et al. 2011; Maharachchikumbura et al. 2014). Nevertheless, based on the results of phylogenetic analyses, *Microdochium*, *Idriella*, and *Selenodriella* were incorporated to a new family introduced as Microdochiaceae by Hernández-Restrepo et al. (2016), which is characterised by asexual morphs that produce polyblastic, sympodial or annellidic conidiogenous cells with hyaline conidia without appendages and sexual morphs that are monographella-like. In *Microdochium*, the color of conidiogenous cells is hyaline, and the shape of conidia seem to be taxonomic important feature. The conidial shape of *Microdochium* is more variable from cylindrical, fusoid or oblong, to lunate, straight or curved, with truncate bases and apices mainly rounded.

Three sections were widely accepted in *Microdochium* based on the type of conidiogenous cells and conidia by Braun (1995) and Hernández-Restrepo et al. (2016). Type I: Microdochium sect. Gerlachia forming annellidic conidiogenous cells with percurrent proliferations; Type II: *Microdochium* forming sympodial, often subdenticulate conidiogenous cells, and relatively more or less fusiform, straight to somewhat curved or falcate, 0–3-septate or even pluriseptate conidia; and Type III: *Gloeocercospora* forming sympodial conidiogenous cells, and very long, scolecosporous and pluriseptate conidia.

From the previous molecular studies of *Microdochium* (Jaklitsch and Voglmayr 2012; Jewell and Hsiang 2013; Zhang et al. 2015), the four gene regions (ITS, LSU, RPB2, TUB2) were chosen in this study. The LSU is informative enough for generic placement of *Microdochium*. The individual gene regions of ITS, TUB2, and RPB2 proved to be able to resolve species in *Microdochium* (results not shown). However, TUB2 was the more informative than other gene regions and showed longer distances between species and higher support values. This results in our study agree with previous studies in other xylariaceous genera (Hsieh et al. 2005; Læssøe et al. 2013; Hernández-Restrepo et al. 2016). By combining phylogenetic analysis and morphology, two species of *Microdochium* were delimited as new species, namely *M. yunnanense* sp. nov. and *M. indocalami* sp. nov. In order to support the validity of these new species, we followed the guidelines of Hernández-Restrepo et al. (2016).

## Supplementary Material

XML Treatment for Microdochium indocalami

XML Treatment for Microdochium yunnanense

XML Treatment for Microdochium
